# Easy and low-cost stable positioning of suspension cells during live-cell imaging

**DOI:** 10.14440/jbm.2017.203

**Published:** 2017-10-17

**Authors:** Daniel Ivanusic, Kazimierz Madela, Joachim Denner

**Affiliations:** Robert Koch Institute, Berlin, Germany

**Keywords:** live-cell imaging, suspension culture, confocal laser scanning microscope

## Abstract

Dynamic processes of cells can be best monitored when living cells are analyzed by imaging. While it is easy to observe adherent living cells it has been extremely challenging to analyze suspension cells. This cell type floats freely in the culture dish, and it is only a question of time when the focus or the observation field is lost. In order to keep the cells in focus, an easy and inexpensive method allowing the observation of living suspension cells during confocal laser scanning microscope imaging was developed.

## INTRODUCTION

**Figure 1.**
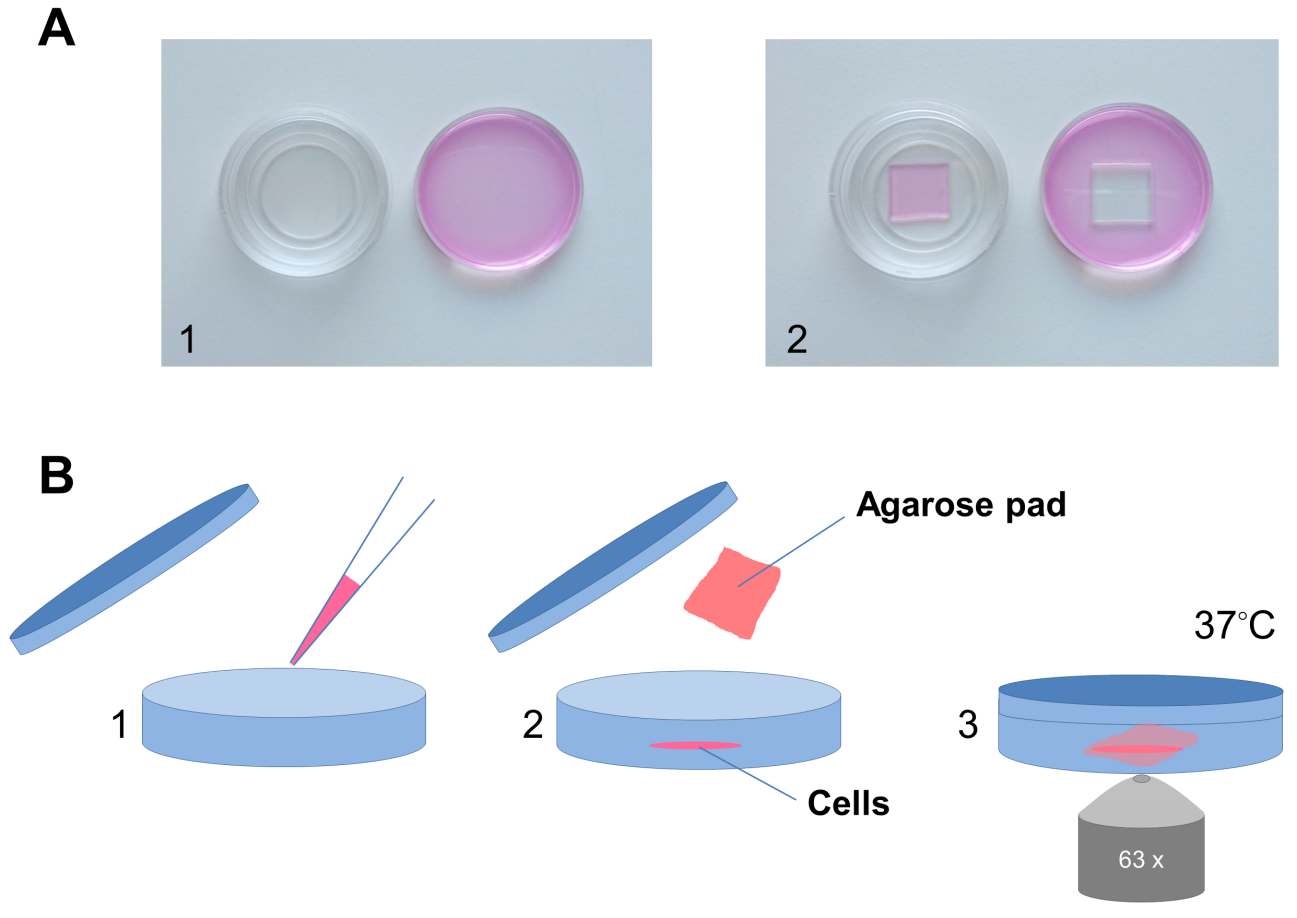
**A.** Preparing the IBIDI dish for live cell imaging of suspension cells. (1) 1 ml of molten agarose in RPMI media was poured into the lid of the IBIDI dish. (2) Approximately 1.2 x 1.2 cm was resected with a scalpel after the agarose was cooled down and placed on top of the cells (not shown) in the bottom of the IBIDI dish. **B.** Schematic presentation of the working steps.

Live-cell imaging techniques combined with confocal laser scanning microscopy (cLSM) enables researchers to reveal mechanisms of protein dynamics and function. The cells need to have a physically stable location in order to avoid free floating through the sample field. Some live-cell imaging applications take up to several hours to obtain images during which time the cells must be permanent in focus. For adherent cells, there is no need to force them into a particular location but when using suspension cells it is impossible to follow up cells in a distinct field. Therefore, a low-cost and easy method to ensure that suspension cells such as Jurkat cells are in a fixed position during live cell imaging was developed. This method is based on the use of an agarose pad in combination with IBIDI cell imaging dishes (**Fig. 1A**). While agarose pads are commonly used for studies of bacteria [1], yeast [2] or *Caenorhabditis elegans* [3], we are not aware of studies on human suspension cells using agarose pads, and inverted microscopes. In this study, the use of agarose pads in combination with suspension cells was shown.

## MATERIALS AND METHODS

### Cell culture and transfection

Jurkat cells were cultured in RPMI medium supplemented with 10% fetal bovine serum (Biochrom AG, Berlin, Germany), 100 IU/ml penicillin, 100 μG/g/ml streptomycin (PAA Laboratories, Cölbe, Germany), and 2 mM L-glutamine (Biochrom AG). Cells were always incubated at 37°C, 5% CO_2_ and a relative humidity of about 95%. Transfection was performed with the plasmid pCMV-CD63-YFP [4] using an AMAXA Nucleofection Kit V (Lonza, **Cologne, Germany**), employing the program S-18 for all transfection procedures and following manufacture’s instructions the day before. Only Jurkat cell cultures in logarithmic growth were used for transfection since this was shown to be crucial for a high transfection efficiency and good cell survival after nucleofection.

### Sample preparation and confocal laser scanning microscopy

The first step is to prepare agarose imaging pads. For this, the RPMI medium without supplements was boiled with 1% agarose of UltraPure quality (Carl Roth, Karlsruhe, Germany). 1 ml of the molten agarose was poured into an IBIDI μ-Dish, 35 mm high (Martinsried, IBIDI, Germany). After the agarose cooled down, a piece of approximately 1.2 × 1.2 cm was cut out with a scalpel (**Fig. 1B**) and placed on the cell suspension applied to the bottom of an IBIDI dish. The thickness of the agarose layer is critical. Whereas agarose pads of 0.5 mm (500 μl molten agarose) can break when applied to the cell suspension, agarose pads of 1 mm (1000 μl molten agarose) were optimal. The height of the agarose pad was calculated by the following equation (1):
(Eqn. 1)
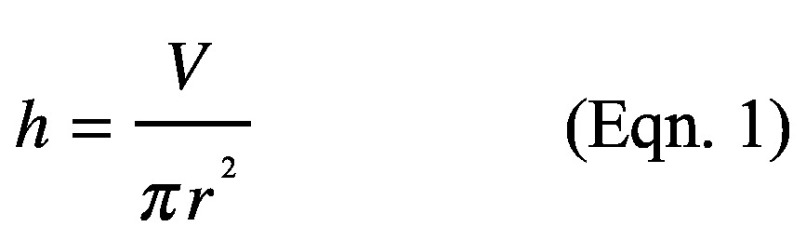

Where *h* is the height of the agarose pad, *V* volume of agarose and *r* the radius of the IBIDI lid.

The cells’ suspension had been cultured and transfected as described above. The volume of the cell suspension added was in the range of 30–50 μl. After covering the cells with the agarose pad the IBIDI dish was closed with a lid (**Fig. 1A**). The IBIDI dish prepared in this way was placed in the prewarmed ZEISS Axio Observer Z1 cLSM 780 microscope with a 63×/1.3 plane Apochromatic objective (ZEISS, Oberkochen, Germany) at an ambient temperature of 37°C. Serial images were obtained to follow up the concentration and distribution of expressed CD63-YFP protein, especially at the basis of filopodia on the cell surface. As control, cells in culture medium without an agarose pad were used. 3D cLSM images were obtained using the Z-stack option and the 3D option in the Zeiss software ZEN 2010 (ZEISS, Oberkochen, Germany). Transmission channel was obtained in the single track mode together with the YFP channel using the excitation laser line at 514 nm.

### Cell viability and cell death analysis

Untransfected Jurkat cells (5 × 10^4^ cells in 50 μL RPMI medium) were as described above applied into an IBIDI dish, covered with an agarose pad and incubated at 37°C. After different time points, the cells were washed from the agarose pad with 450 μL ViaCount reagent (Milipore, Darmstadt, Germany) in order to generate a single cell suspension. The number of viable cells at each time point was assayed using the flow cytometer Guava Personal Cell Analysis (PCA) System (Milipore, Darmstadt, Germany). As controls cells not incubated under an agarose pad and cells not incubated under an agarose pad, but treated with 1% Triton X-100 (Carl Roth, Karlsruhe, Germany) were used. Cell membrane defects were assessed by measuring the lactate dehydrogenase (LDH) release in the cell supernatants at each time point. IBIDI dishes with Jurkat cells were prepared as described for the cell viability assay and to the culture medium in the IBIDI dishes 400 μL PBS was added. The mixture was collected and centrifuged in order to separate the cells, and 300 μl of the supernatant from each time point were used in triplicates to measure the level of LDH release with the Roche Cytotoxicity Detection Kit (Roche Diagnostics, Mannheim, Germany). The optical density was measured at 492 nm using a microplate reader Thermo Multiskan GO (*Thermo* Fisher Scientific, *Vantaa*, Finland). As a control, total LDH release from cells treated with 1% Triton X-100 was measured (Carl Roth, Karlsruhe, Germany).

## RESULTS AND DISCUSSION

**Figure 2.**
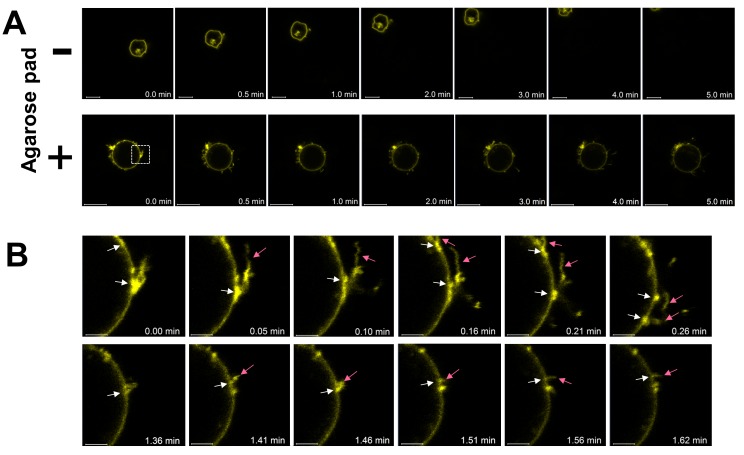
Live-cell imaging with and without agarose pads. Images of transfected Jurkat cells expressing the protein CD63-YFP were obtained. **A.** The cell without an agarose pad (-) is able to float freely through the IBIDI dish, whereas the cells with agarose pad (+) cannot. The time is given in minutes, scale bars 10 μm. **B.** Filopodia extended predominantly from the CD63-YFP enriched basis. White arrows point to a very high CD63-YFP protein concentration at the point from where the filopodia are extending (pink arrows), the time is given in minutes, scale bars 2 μm.

Using agarose pads, images were obtained under stable conditions without any floating effects (**Fig. 2A**) and the selected cells covered with an agarose pad were always in focus. Standard fixation of mammal cells with poly-L-Lysine coating during live-cell imaging [5-7] was no longer needed when an agarose pad was applied to the cells. Coating dishes with poly-L-lysine was supposed to act by electrostatic interactions between the coated dish and the cell membrane [8]. The use of agarose pads reduced the level of non-specific binding to a minimum in life-cell experiments. This method not only keeps the cells fixed in focus and prevents their accidental movement, but also provides cells with a significant volume of culture media to ensure cell viability when compared with samples without an agarose pad (**Fig. 2A**). In order to validate our method, the expression of CD63-YFP at the plasma membrane of transfected Jurkat cells was monitored. It was shown that the CD63-YFP protein was concentrated at the filopodia basis formation on the plasma membrane. We observed that the filopodia extended predominantly from the CD63-YFP enriched basis (**Fig. 2B**). The ability of cells to migrate strongly depends on a dynamic actin assembly [9] and the protein superfamily of tetraspanins such as CD63 was shown to be involved in cell migration and *adhesion* [10]. Other dynamic entities which co-cluster in the membrane with CD63-YFP can also be studied with this new method. For example, the stable focusing and positioning of cells enable excellent images for time-lapse videos.

**Figure 3.**
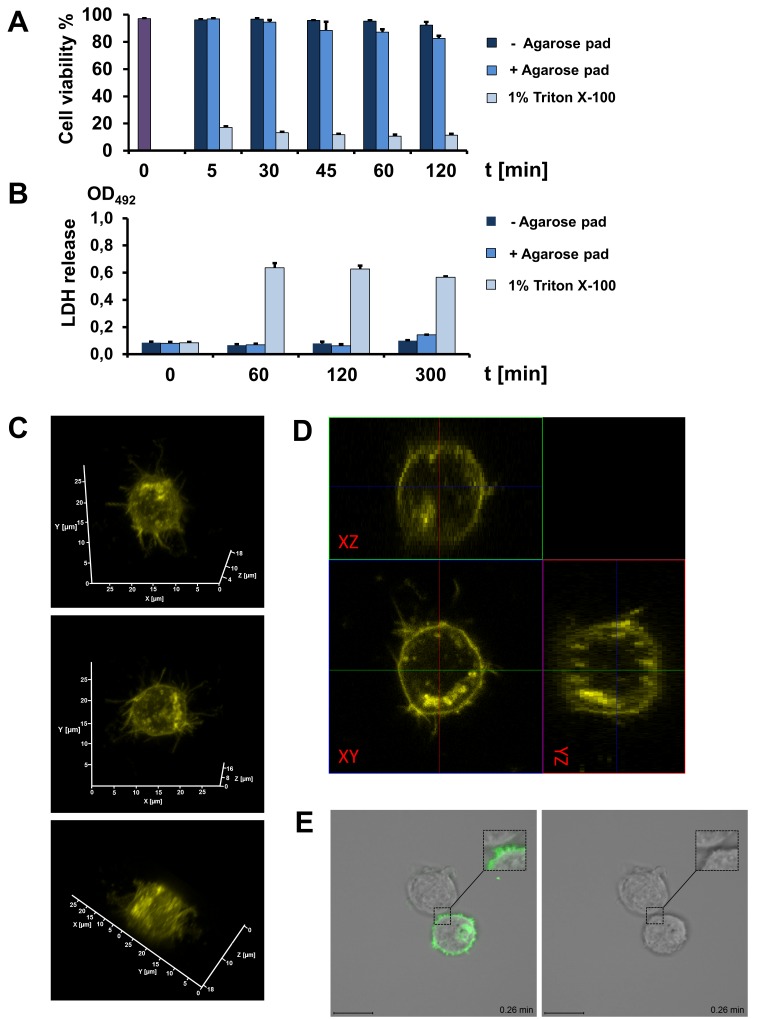
Influence of the agarose pad on cell viability, cytotoxicity, cell shape and transmission channel. **A.** Viability of the Jurkat cells was measured using the Guava PCA ViaCount assay. **B.** Supernatants from cultured Jurkat cells were analyzed for LDH release using an LDH colorimetric *assay*. The results from the Guava flow cytometry and OD values are presented as the mean ± standard deviation for triplicates at each time point. **C.** Images of a 3D reconstruction from z-stack sections of CD63-YFP-expressing Jurkat cell under an agarose pad. The 3D cell shape was virtually rotated and different axis views of the reconstruction were displayed. **D.** CLSM orthogonal projections of z-stacks images taken from a Jurkat cell expressing the protein CD63-YFP. The side top panel (xz-direction) and to the right panel (yz-direction) indicate a projection from the attachment of the agarose pad to the IBIDI dish surface. **E.** Images of Jurkat cells expressing CD63-YFP. The cells were cultured using an agarose pad and in addition, the YFP channel and the transmission channel was obtained, scale bar 10 μm.

In order to investigate putative cytotoxic effects of the applied agarose pad on the cell suspension, a flow cytometry based viability testing [11] and a measurement of lactate dehydrogenase (LDH) release as a marker for membrane damage [12,13] were performed. Incubation of Jurkat cell for 2 h with an agarose pad showed a viability of over 87% while cells without an agarose pad had a viability of 95% (**Fig. 3A**). The release of LDH was slightly higher from cells covered with an agarose pad after 5 h (**Fig. 3B**). These data show that most of the cells are viable during live-imaging within a 2 h window. We can exclude that cells were flattened by the applied agarose pad using a three dimensional (3D) cLSM image reconstruction (**Fig. 3C**) and orthogonal projections (**Fig. 3D**). The round 3D shape of the cell can clearly be seen and is characteristic for Jurkat cells. As well, the method using agarose pads does not influence the brightness of the transmission channel. Studying cell-cell contacts between Jurkat cells transmission images could be taken without any interference from the agarose pad (**Fig. 3E**). In summary, using agarose pads improved the conventional imaging techniques when working with suspension cells. This low-cost technique is simple to apply and rapid, and helps to achieve a stable positioning for live-cell imaging experiments.
